# Detection of Extended-Spectrum Beta-Lactamase-Producing* Escherichia coli* in Market-Ready Chickens in Zambia

**DOI:** 10.1155/2016/5275724

**Published:** 2016-04-17

**Authors:** K. Chishimba, B. M. Hang'ombe, K. Muzandu, S. E. Mshana, M. I. Matee, C. Nakajima, Y. Suzuki

**Affiliations:** ^1^Lusaka City Council, Public Health Department, P.O. Box 30789, Lusaka, Zambia; ^2^Microbiology Unit, Paraclinical Studies, University of Zambia, P.O. Box 32379, Lusaka, Zambia; ^3^Weill Bugando School of Medicine, Catholic University of Health and Allied Sciences, P.O. Box 1464, Mwanza, Tanzania; ^4^Department of Microbiology and Immunology, School of Medicine, Muhimbili University of Health and Allied Sciences, P.O. Box 65001, Dar es Salaam, Tanzania; ^5^Division of Bioresources, Hokkaido University Research Center for Zoonosis Control, Kita-20, Nishi-10, Kita-ku, Sapporo 001-0020, Japan; ^6^The Global Station for Zoonosis Control, Hokkaido University, Sapporo, Kita-20, Nishi-10, Kita-ku, Sapporo 001-0020, Japan

## Abstract

The frequent administering of antibiotics in the treatment of poultry diseases may contribute to emergence of antimicrobial-resistant strains. The objective of this study was to detect the presence of extended-spectrum *β*-lactamase- (ESBL-) producing* Escherichia coli* in poultry in Zambia. A total of 384 poultry samples were collected and analyzed for ESBL-producing* Escherichia coli*. The cultured* E. coli* isolates were subjected to antimicrobial susceptibility tests and the polymerase chain reaction for detection of *bla*_CTX-M_, *bla*_SHV_, and *bla*_TEM_ genes. Overall 20.1%, 77/384, (95% CI; 43.2–65.5%) of total samples analyzed contained ESBL-producing* Escherichia coli*. The antimicrobial sensitivity test revealed that 85.7% (66/77; CI: 75.7–92) of ESBL-producing* E. coli* isolates conferred resistance to beta-lactam and other antimicrobial agents. These results indicate that poultry is a potential reservoir for ESBL-producing* Escherichia coli*. The presence of ESBL-producing* Escherichia coli* in poultry destined for human consumption requires strengthening of the antibiotic administering policy. This is important as antibiotic administration in food animals is gaining momentum for improved animal productivity in developing countries such as Zambia.

## 1. Introduction

Extended-spectrum beta-lactamase (ESBL) producers are Gram-negative bacteria that produce enzymes that bestow resistance to most beta-lactam antibiotics like penicillins, cephalosporins, and the monobactam aztreonam [1]. These ESBL producers have been noticed mainly in the Enterobacteriaceae family of bacteria which may harbour several antibiotic resistance determinants making treatment of infections caused by these pathogens more difficult [2].

Extended-spectrum beta-lactamase producers have a complex epidemiology; the most prominent bacteria involved include* E. coli* and* K. pneumoniae* whose reservoirs comprise the environment (soil and water), wild animals, farm animals, food, and pets [3]. In some communities, backyard poultry houses at residential premises may disseminate antimicrobial-resistant ESBL producers in the environment as previously observed [4]. A study conducted in Spain identified the presence of ESBL-producing bacteria in poultry with* E. coli* strains comprising ESBL CTX-M-14, CTX-M-9, and SHV-12 [5]. In another study conducted in Britain, 54.5% of CTX-M-carrying* E. coli* was isolated from broiler abattoirs and 3.6% from individual broiler caecal samples [6]. Food animals colonized with ESBL-producing bacteria can enhance the spread of bacteria at the community level [7]. Antimicrobial-resistant* E. coli* asymptomatically colonizes the intestinal flora of food animals with a likelihood of becoming infectious to humans if consumed through the food chain [8]. Furthermore the predominant presence of Cefotaxime-Munich genes in healthy broiler chickens can in the long run spread the resistant ESBL-genes from the rearing environment into the food chain [3]. Contamination of meat products during slaughtering, dressing, and evisceration of internal contents is another risk factor that can result in further spread of resistant ESBLs genes within the human population [3, 6].

Extended-spectrum beta-lactamase-producing bacteria are frequently resistant to many antimicrobial agents usually recommended for the treatment of infections such as gentamicin, fluoroquinolones, and trimethoprim-sulfamethoxazole [9]. This leads to serious challenges in the treatment of ESBL-Enterobacteriaceae infections because the bacterial plasmid may harbour several antibiotic resistance determinants [2]. Heavy usage of antibiotics has been reported to be a risk factor in the acquisition of ESBL-producing organisms. An increased trend of resistance to commonly used antibiotics, namely, ampicillin, cotrimoxazole, gentamicin, erythromycin, tetracycline, and third-generation cephalosporins, has been observed [10, 11].

In the present study we found the presence of ESBL-producing* E. coli* bacteria in market-ready chickens. These organisms have been established to confer resistance to many antimicrobial agents recommended for the treatment of infections such as gentamicin, fluoroquinolones, and trimethoprim-sulfamethoxazole [2]. This leads to serious challenges in the treatment of ESBL-Enterobacteriaceae infections [10, 11]. A baseline study was therefore initiated to determine the carriage of ESBL in food of animal origin in market-ready broiler chickens in Zambia.

## 2. Materials and Methods

### 2.1. Study Area and Sampling

The study was conducted in Lusaka, the capital city of Zambia. Lusaka covers an estimated area of 360 km^2^ and is located at 15°30′ latitude south and 28°17′ longitude east. The city lies on a plateau 1280 m above sea level [12]. A poultry abattoir was considered for the purpose of poultry swab sampling at a point where broiler chickens were to enter the market. A total of 384 poultry samples were collected at the slaughterhouse, before the carcasses were chilled.

The swabs were labelled appropriately and collection of the swab samples was aseptically done. This involved the use of sterile swab sticks (Oxoid, Basingstoke, UK) which were placed in tubes containing a Cary-Blair transport medium (Oxoid, Basingstoke, UK). The poultry samples were inoculated on MacConkey agar (Oxoid, Basingstoke, UK) containing 2 mg/L of cefotaxime (Sigma-Aldrich, Munich, Germany) for preliminary screening of ESBL-producing bacteria [13]. The plates were later incubated at 37°C for 24 hours. The colonies that grew on MacConkey agar were identified as lactose fermenters or nonlactose fermenters. Identification of* E. coli* lactose-fermenting positive colonies was done using phenotypic characteristics and confirmed by the Triple Sugar Iron (TSI) and IMViC tests as described by Rayamajhi et al. [13] and Batchelor et al. [14]. For genetic detection,* E. coli* isolates were cultured on brain-heart-infusion broth (Nissui, Tokyo, Japan) at 37°C for 24 hours. After incubation, DNA was extracted by boiling methods [15]. The* E. coli* isolates were subjected to PCR for confirmation of resistance genes: TEM (Temoniera), SHV (Sulphydryl Variable), and CTX-M (Cefotaxime-Munich) using primers previously used by other workers [14, 16]. The PCR (Finnzymes Oy, Finland) was performed in a total reaction volume of 10 *µ*L consisting of 5 *µ*L Phusion master mix, 2 *µ*L sterile distilled water, 2 *µ*L primers (forward and reverse), and 1 *µ*L bacterial DNA template. The PCR was performed using the rapid cycle DNA amplification method comprising an initial denaturation step at 98°C for 30 seconds, followed by 35 cycles of template denaturation at 98°C for 1 second and primer annealing at 60°C for 5 seconds and 72°C for 1 second with final extension at 72°C for 10 seconds. The PCR products were later viewed with ethidium bromide after electrophoresis through 1.5% agarose gel [17].

### 2.2. Antibiotic Sensitivity Testing

The antimicrobial susceptibility testing was done using the Kirby-Bauer disc diffusion method on Mueller Hinton Agar (Becton, Dickinson and Company, MD, USA) based on the Clinical Laboratory Standard Institute (CLSI) guidelines [18]. The antibiotic discs (Becton, Dickinson and Company, MD, USA) used included ampicillin (10 *μ*g), sulfamethoxazole/trimethoprim (1.25/23.75 *μ*g), streptomycin (300 *μ*g), ciprofloxacin (5 *μ*g), tetracycline (30 *μ*g), gentamicin (10 *μ*g), nalidixic acid (30 *μ*g), chloramphenicol (30 *μ*g), ceftazidime (30 *μ*g), norfloxacin (10 *μ*g), and cefotaxime (30 *μ*g). The phenotypic confirmation of ESBL isolates was done by the combination of disc approximation method using either ceftazidime (30 *μ*g) or cefotaxime (30 *μ*g) alone followed by overnight incubation at 37°C for 18–24 hrs. Interpretation of susceptibility patterns on other antimicrobial discs was done using guidelines laid down in the CLSI, which provides break points corresponding to zone of inhibition diameter. An increase in antibiotic zone diameter (5–12 mm) for either ceftazidime or cefotaxime indicated ESBL production [10, 18]. Quality control standard laboratory procedures were strictly adhered to, to avoid contamination.* Escherichia coli* ATCC 25922 was used as a quality control organism.

## 3. Results

Of the 384* E. coli* isolates analyzed for the presence of ESBL-producing isolates on PCR, a total of 20.1%, 77/384, (95% CI; 43.2–65.5%) were confirmed to be ESBL-producing isolates. The ESBL-producing* E. coli* isolates carried the *β*-lactamase genes of either *bla*_CTX-M_, *bla*_SHV_, and *bla*_TEM_ or a combination. The major gene detected on PCR was *bla*_CTX-M_ cluster, followed by *bla*_SHV_ cluster, and *bla*_TEM_ cluster. A combination of *bla*_CTX-M_ + *bla*_TEM_ cluster showed 4.4% (17 isolates). This was followed by a combination of *bla*_CTX-M_ + *bla*_SHV_ cluster and *bla*_CTX-M_ + *bla*_SHV_ + *bla*_TEM_ cluster with 0.52% (2 isolates) and the lowest was a combination of *bla*_TEM_ + *bla*_SHV_ cluster with 0.3% (1 isolate) (Table 1).

The seventy-seven (77)* E. coli* isolates preliminarily identified as ESBL-producing* E. coli* were subjected to antibiotic susceptibility testing. Of 77* E. coli* isolates analyzed, 85.7% (66/77; CI: 75.7–92) were resistant to one or several antimicrobial compounds. The diversity of the antibiotic resistant and susceptible* E. coli* isolates was clearly observed with 66 isolates being resistant to one or more antibiotics used and 11 isolates being susceptible to all antibiotics tested. Interestingly, high resistance rates were noticed in ampicillin (100%), cefotaxime/ceftazidime (100%), tetracycline (59.7%), chloramphenicol (57.1%), and norfloxacin (54.5%) (Table 2). It was further observed that multidrug resistance (MDR) of* E. coli* isolates to six or more drugs was most frequent (45.5%, 35/77) followed by resistance to five drugs (11.7%, 9/77) and four drugs (11.7%, 9/77). Based on the antimicrobial susceptibility test results,* E. coli* from poultry in this study can be grouped in six phenotypes (Table 3).

## 4. Discussion

Bacteria in food-producing animals are spread through the food chain. In Africa, the desire to optimize animal production has led to indiscriminate use of antibiotics. The heavy usage of antibiotics has been reported to be a risk factor for acquisition of ESBL-producing organisms resulting in an increased trend of resistance to commonly used antibiotics, namely, ampicillin, cotrimoxazole, gentamicin, erythromycin, tetracycline, and third generation cephalosporins [16]. In Zambia, drugstores which stock human and livestock antimicrobial agents are coming up without proper guidance on the dispensation of drugs. The inadequate monitoring of the drugstores in the nation has led people to easily access the livestock antibiotics without following the recommended laid down clinical prescription procedures thereby increasing the risk of ESBL in food animals. Detection of ESBL-producing* E. coli* isolates in poultry has not been fully conducted in Zambia. This cross sectional study revealed that an overall of 20% ESBL-producing* E. coli* isolates were detected in poultry. The high prevalence observed may be due to frequent administration of antimicrobials to poultry which in turn increases the risk of higher antimicrobial-resistant* E. coli* strains in the normal intestinal flora as observed in a study conducted by Carattoli [3]. In this study, it was established that food animals were possible reservoirs for resistant faecal flora particularly* E. coli.* Furthermore, the high colonization rate could be attributed to cross contamination of poultry in abattoirs particularly during slaughtering and dressing. The processes of slaughtering and dressing are potential risk factors that may exacerbate the transmission rate of ESBL-producing* E. coli* resistant genes as described by Lavilla et al. [8] and Reich et al. [16].

The predominant ESBL-producing* E. coli* gene identified in this study was *bla*_CTX-M_ cluster with 13% which was relatively low in relation to 54.5% of *bla*_CTX-M_ carrying* E. coli* isolated from poultry chickens in Britain [6]. In Zambia, the economic situation has led many residents to rearing broiler chickens as an income generating venture. This brings humans into close contact with poultry which could be a possible route of shedding ESBL producers into the environment. However, no detailed studies have yet been conducted in Zambia to specifically describe the types of ESBL-producing Enterobacteriaceae present in apparently healthy chickens and other food animals.

In addition, the study also showed that ESBL-producing* E. coli* isolates carried multiple types of beta-lactamase genes and that the combination of *bla*_CTX-M_ + *bla*_TEM_ cluster was predominant followed by *bla*_SHV_ cluster and *bla*_TEM_ cluster. This therefore confirms that ESBL-mediated plasmids are capable of carrying more than one beta-lactamase gene and thus result in high-level presence of beta-lactam resistant phenotypes as described by Rottier et al. [2]. Previous studies in Spain identified* E. coli* strains comprising *bla*_CTX-M-14_, *bla*_CTX-M-9_, and *bla*_SHV-12_ as the predominant ESBL-producing subtypes isolated among poultry [12].

Antimicrobial susceptibility testing revealed interesting patterns with resistance rates observed in the majority of antimicrobial agents tested. From the antimicrobial susceptibility results, 85.7% (66/77; CI: 75.7–92) of isolates were resistant to chloramphenicol, ciprofloxacin, gentamicin, nalidixic acid, norfloxacin, streptomycin, sulfamethoxazole/trimethoprim, and tetracycline. The findings are similar to studies conducted by Mshana et al. [10]. For instance, a study on published literature for Democratic Republic of Congo, Mozambique, Tanzania, and Zambia revealed an increased trend of resistance to ampicillin, cotrimoxazole, gentamicin, erythromycin, tetracycline, and third-generation cephalosporins [11]. In this study, high resistance rates to beta-lactam drugs, namely, ampicillin (100%), cefotaxime, and ceftazidime (100%), were observed among the isolates investigated. At least six phenotypes were observed from this study, indicating a wide diversity of resistance. Unfortunately, the study had a limitation as it was not able to establish the type of antimicrobials frequently used for poultry feed and growth promotion.

## 5. Conclusion

This is the first study to be conducted in Zambia on detection of ESBL-producing* E. coli* isolates in poultry. This therefore further confirms that poultry can be one of the major and potential reservoirs for the antimicrobial ESBL resistant genes which could spread into the food chain. The study has also shown a widespread occurrence of multidrug resistance patterns of* E. coli* isolates in poultry.

## Figures and Tables

**Table 1:** Confirmed ESBL-producing *E. coli* (using PCR and gene detection) isolates.

Detected gene (s)	Number of *E. coli* isolates	% of *E. coli* isolates (*n* = 384)^a^
SHV	2	0.52
CTX-M	50	13
TEM	3	0.8
CTX-M and TEM	17	4.4
CTX-M and SHV	2	0.52
TEM and SHV	1	0.3
CTX-M, SHV, and TEM	2	0.52
None^b^	307	79.9
Proven ESBL producers	77	20.1

^a^384 *E. coli* isolates suspected of being ESBL producers were examined.

^b^Negative in all PCRs.

**Table 2:** Antibiotic susceptibilities of the isolates *n* = 77.

Antibiotic	Resistant number (%)
Cefotaxime/ceftazidime	77 (100%)
Ampicillin	77 (100%)
Chloramphenicol	44 (57.1%)
Ciprofloxacin	37 (48.1%)
Gentamicin	29 (37.7%)
Nalidixic acid	37 (48.1%)
Norfloxacin	42 (54.5%)
Streptomycin	15 (20.8%)
Sulfamethoxazole/trimethoprim	32 (41.6%)
Tetracycline	46 (59.7%)

**Table 3:** Antibiotic resistance patterns of *E. coli* isolates.

Antibiotic combination	Number of resistant isolates	%	Observation
Ampicillin and tetracycline	1	1.3	Resistant to two antibiotics

Ampicillin, tetracycline, chloramphenicol, and ceftazidime	3	3.9	Resistant to four antibiotics

Ampicillin, ceftazidime, chloramphenicol, and norfloxacin	3	3.9	Resistant to four antibiotics

Ampicillin, chloramphenicol, cefotaxime, and sulfamethoxazole	3	3.9	Resistant to four antibiotics

Ampicillin, streptomycin, gentamicin, tetracycline, and cefotaxime	9	11.7	Resistant to five antibiotics

Ampicillin, streptomycin, tetracycline, cefotaxime, nalidixic acid, ceftazidime, norfloxacin, and ciprofloxacin	35	45.5	Resistant to six antibiotics

## Abbreviations

ESBL: Extended-spectrum beta-lactamases
PCR: Polymerase chain reaction.
